# Connecting communities across the globe: Atlas protocol

**DOI:** 10.1177/26323524251396994

**Published:** 2025-12-04

**Authors:** Rebecca Newell, Juan Esteban Correa-Morales, Vilma A. Tripodoro, Steven Vanderstichelen, Ghauri Aggarwal, Samar Aoun, Erin Das, Farah Demachkieh, James Downar, Silvia Librada, Julieanne Hilbers, Julie Lapenskie, Emmanuel Luyirika, Saif Mohammed, Masanori Mori, Ekkapop Sittiwantana, Libby Sallnow

**Affiliations:** 1 Public Health Department, Brighton and Sussex Medical School, UK; 2 Marie Curie Palliative Care Research Department, University College London, UK; 3 ATLANTES Global Observatory of Palliative Care, Institute for Culture and Society, University of Navarra, Pamplona, Spain; 4 Instituto Pallium Latinoamérica, Buenos Aires, Argentina; 5 End-of-Life Care Research Group, Vrije Universiteit Brussel, Belgium; 6 Compassionate Communities Centre of Expertise (COCO), Vrije Universiteit Brussel, Belgium; 7 Asia Pacific Hospice Palliative Care Network, National Cancer Centre Singapore; 8 University of Western Australia, and Perron Institute, QEII Medical Centre, Nedlands, WA, Australia; 9 Compassionate Communities Australia; 10 Global Treehouse Foundation, London, UK; 11 Palliative Care in Humanitarian Aid Situations and Emergencies (PallCHASE), Edinburgh, Scotland; 12 SANAD: The Home Hospice Organization of Lebanon, Beirut, Lebanon; 13 Pan-Canadian Palliative Care Research Collaborative, Ottawa, ON, Canada; 14 New Health Foundation, Sevilla, España; 15 African Palliative Care Association, Kampala, Uganda; 16 World Health Organisation Collaborating Centre, Institute of Palliative Medicine, Kozhikode, India; 17 The Peaceful Death Project, Bangkok, Thailand

**Keywords:** compassionate communities, palliative care, community engagement, public health palliative care, primary palliative care

## Abstract

**Background:**

Providing universal access to palliative care is increasingly recognised as a global public health priority, especially in low- and middle-income countries. Compassionate communities could help with provision by fostering community-led responses to dying, death and grief. Despite their global growth, compassionate communities are often absent from national and international palliative care strategies, and few are represented in academic literature. This, along with their diversity and community-defined nature, can contribute to difficulties in documentation, evaluation and visibility, limiting the ability to showcase their benefits, form partnerships and evaluate practice.

**Objectives:**

This study protocol outlines the development of the first global atlas of compassionate communities, aiming to map their locations and increase understanding of their structures, activities and impacts.

**Design:**

A participatory methodology was used. This enabled global participants to complete and share the survey based on their experiences with community programmes addressing serious illness, dying and grief. A diverse steering committee guided the design, validation and piloting of the survey to ensure clarity, cultural sensitivity and accessibility. Eighteen global experts contributed to developing and validating the survey, with 14 of 15 items meeting the Content Validity Index threshold. The final survey captures data on location, aims, evaluation, challenges and impact. Dissemination involves global networks, social media and snowball sampling.

**Discussion:**

This protocol addresses a critical gap in Public Health Palliative Care literature by providing an inclusive and participatory method to map the compassionate community’s landscape. The resulting data will promote visibility, partnerships and future research, supporting greater recognition of global compassionate communities and their contributions to primary palliative care.

## Plain language summary

Creating a global map of compassionate communities supporting palliative care

This study shares a plan to create the first global map of compassionate communities—local groups that help people through serious illness, dying, and grief. A simple, culturally sensitive survey was developed with input from global experts to collect information on where these communities are, what they do, and how they make a difference. The goal is to raise awareness, build partnerships, and support future research to show how these community efforts improve palliative care around the world.

## Background

The World Health Organization recognises palliative care as a key priority to improve the quality-of-life and reduce suffering for individuals with life-limiting illnesses and their families, especially in low- and middle-income countries.^1–6^ Central to achieving this goal is primary palliative care, commonly defined as generalist palliative care delivered by healthcare providers such as general practitioners within the community. Specialised palliative care, on the other hand, typically involves multidisciplinary teams including physicians, nurses, social workers and other professionals with advanced training in managing complex symptoms, psychosocial and spiritual needs. These teams provide specialist-level care, in an inpatient setting, as well as support in the community, collaboratively and complementing generalist providers to ensure integrated, patient-centred care.^1,7,8^

However, the distinction between primary and specialist palliative is often blurred and can vary across different global contexts. In some contexts, specialist teams provide community-based primary palliative care, whilst in others, specialist palliative care services may be limited or non-existent, with generalist providers assuming both roles. We will be defining primary palliative care, as both generalist palliative care, but also specialist-level care supported within the community.^7^

Public health palliative care adopts a broader framework, integrating efforts by social, civil and healthcare actors to address palliative care needs holistically.^3,8^ This approach is grounded in the Ottawa Charter, emphasising the enhancement of population well-being around death and dying through community action, individual empowerment, supportive environments and integration with health systems.^4,9–12^ It has inspired community-based participation, most notably seen in the emergence of compassionate communities or compassionate cities.^9^ These initiatives harness collective compassion to empower individuals, groups and societal structures to mitigate or prevent harm associated with serious illness, death, dying and grief, while recognising these experiences as integral parts of everyday life.^5,9,13–15^

Since their emergence in the early 2000s, compassionate communities have grown substantially and are now found globally.^11^ Their public health relevance is underscored by several factors. They can promote views of serious illness, care, death and grief as a collective duty and social process. They can cultivate death literacy as a vital tool for empowering individuals and communities.^16,17^ Compassionate communities, through the nature of community action, often intersect with social determinants of health, for example, housing, education and workplaces, and this can lead to initiatives addressing these, either intuitively or through deliberate intent.^18,19^ They can promote social connectedness, improve health and quality-of-life, impact caregiver burden and reduce social isolation.^20,21^ In addition, compassionate communities have demonstrated cost-effectiveness by reducing the burden on healthcare systems, optimising resource utilisation and minimising reliance on emergency department visits and out-of-hours healthcare consultations.^4,5,9,11,12,17,22,23^

However, as compassionate communities emerge from a range of contexts, tailored to specific local circumstances, and individuals’ or caregivers’ illness trajectories and needs, they exhibit significant heterogeneity. This diversity has made it challenging to establish a comprehensive yet specific definition, as well as to determine how many compassionate communities exist and how to measure their impact.^9,11,24,25^ This lack of comprehensive data has limited the ability to showcase their benefits, expand their adoption and develop frameworks to promote implementing this public health palliative care strategy.^24^

Despite their global growth, compassionate communities are often absent from national and international palliative care strategies, and few are represented in academic literature.^1,26,27^ A 2022 systematic review by Quintiens et al. identified 26 compassionate communities but found that few initiatives were properly evaluated, with many assessments misaligned with their intended goals.^10^ Another recent scoping review on the assessment of compassionate communities revealed that only 30% measure their impact, and within that 88% were analysed at the individual level, rather than focusing on community processes and outcomes.^24^ These community-based initiatives are not easily measured, due to their complexity, heterogeneity and a challenge with measuring broader social impacts using conventional healthcare metrics, which typically focus on healthcare access or clinical outcomes at an individual level.^10,24,28^

While these reviews provide an overview, tracking compassionate communities through academic literature has significant limitations. Publishing in peer-reviewed journals is often time-consuming, costly and hindered by language barriers—factors that communities may not prioritise, as academic dissemination may not align with their goals or needs.^29^ As a result, there is limited data on the number and locations of these and a significant gap in understanding the broader social and emotional impacts of these initiatives.^24,30^ Abel and Kellehear argue that to address this broader understanding of Public health palliative care (PHPC) approaches, we will need new methodologies, epistemologies and interdisciplinarity of partners, to decentralise our current understandings and see beyond them.^28^

Overcoming this knowledge gap is crucial. Firstly, from a theoretical perspective, to avoid a skewed image of reality and biased sample, and secondly, from a practical perspective to avoid creating models that do not work in context or lose their grounding in practice. Bias samples and the potential for frameworks that underrepresent certain populations pose questions on epistemic justice and power. Eurocentric frameworks have traditionally dominated this field, with decolonisation asking us to critically examine the historical, social, political context of how we create and value knowledge from different sources.^31^ The experiences of death, dying, grief and suffering are rooted in the social and cultural contexts.^32^ Valuing and incorporating knowledge from a range of voices, for example from, indigenous or traditional knowledge as highlighted by Synder, can promote equity and promote care that is more responsive to the needs of diverse communities.^33,34^

Understanding the number and locations of compassionate communities is a step towards achieving this broader understanding. Increased awareness will enable more effective advocacy, facilitate the sharing of their impact and create opportunities for partnerships. In response to this need, and with the goal of diversifying the representation of compassionate communities and capturing their heterogeneity and unique perspectives, this research aims to create the first global atlas of compassionate communities.^35^

## Aims

1. To map compassionate community initiatives to create a global atlas, documenting their location, aims, partnerships and evaluation methods.
2. Identify global patterns across compassionate communities based on region, sector and demographics.
3. Explore the global scope and diversity of initiatives.

## Design

This is a quantitative survey study using a convenience and snowballing sampling method, and applying a citizen science approach.

### Community-engaged

Individuals from around the world will contribute through survey dissemination and data collection based on their lived experiences with compassionate communities. This utilises aspects of a citizen science approach, with the aim of engaging and enabling a broad population to collect and report data via a survey, based on their personal experiences with compassionate communities. Citizen science, which involves the active participation of the general public in scientific research through contributing their knowledge, resources and skills to collect or analyse data, has proven to be an innovative and effective methodology for promoting health equity in public health.^36–39^ It does so by engaging communities to define problems, mobilise resources and translate findings into high-impact solutions.^38,39^ This study adopts a ‘citizen science with the people’ approach, involving public participation in standardised data collection.^36,40^

### Concepts

Although the label *compassionate communities/cities* has gained broad recognition and has existed for over two decades, the term may still be unfamiliar in many cultures or societies, where alternative mechanisms may be used to support processes of death, dying and grief. The diverse and creative ways in which communities foster compassion often extend beyond the boundaries set by existing theoretical frameworks.^11,24,25^

The aim of this study was not to impose a definition of compassionate communities, but rather to allow participants to define them based on their experiences of building or participating in compassionate communities, allowing them to offer and expand our current understanding of what compassionate communities are. Nonetheless, a basic conceptual foundation was necessary for meaningful data collection. Therefore, to avoid limiting participation to those familiar with the term ‘compassionate communities’, the study avoided solely using this label. Instead, a broad and accessible definition—adapted from Vanderstichelen et al. and one of the most widely accepted in the field—was provided to participants (see Table 1). The language was simplified to ensure accessibility across diverse populations and language barriers, with translations into a wide range of global written languages.^14^ The primary criterion for inclusion is the community’s self-perception and the extent to which its initiative enhances experiences related to death, dying and grief in a community setting (Table 2). Only current communities will be included. In cases of unclear alignment or difficult classification, researchers will deliberate with a diverse steering committee, assessing if the initiative fits with a health promotion approach to community-based primary palliative care, with decisions documented and reported in the analysis.

**Table 1.** Definition provided through the survey.

Community-based initiatives that support people through illness, caregiving, dying and grief—often referred to as compassionate communities. A community programme is any organised effort within a community that helps people with experiences of illness, caregiving, death, dying and grief. These can take many forms, such as support groups, awareness campaigns, or educational.

**Table 2.** Community working criteria for inclusion.

- Compassionate communities are social models of care that recognise serious illness, death, dying and loss as everyday aspects of life. - They aim to prevent or reduce the harms associated through these at a community level using a holistic approach. - They promote individual behaviour, group strategies or societal structures or policies that prevent or reduce suffering resulting from experiences of serious (mental or physical) illness, death, dying and loss. - They actively promote health and well-being, community support and empowerment of community members affected by such experiences - They should be currently active communities.

### Survey design

To ensure the validity, relevance and cultural acceptability of the survey, a diverse steering committee composed of global experts in community-based palliative care has been established. This group was created with the aim to reflect a breadth of perspectives, with members representing a range of world regions (including Africa, Asia, Latin America, Europe and Oceania), genders and backgrounds from clinical and public health expertise to community activism and lived experience.

A draft set of survey questions developed by the core research group, which includes three leading organisations in this area—ATLANTES Global Observatory of Palliative Care–WHO Collaborating Centre, Public Health Palliative Care International (PHPCI) and the Compassionate Communities Centre of Expertise—that are actively engaged in mapping community-based primary palliative care research. The initial question set was informed by relevant literature^9,10,13,14,24^ and the work of González-Jaramillo et al., which highlights the benefits of community-based primary palliative care in three countries.^30^

The draft survey was then refined through cognitive interviews conducted with members of the steering committee. Cognitive interviewing involves participants verbalising their thoughts while completing the survey, a method known to be effective in identifying ambiguities, improving cultural sensitivity and translation, and enhancing both the reliability and completeness of responses.^41,42^ This technique is especially valuable when translating surveys and exploring novel health concepts. The feedback includes qualitative insights into issues, such as misinterpretation of terms, ambiguous wording, cultural mismatches, emotional discomfort and challenges with response options.^41–43^ Quantitative evaluation will be conducted using the Cognitive Validity Index, which assesses each item’s clarity, relevance, cultural appropriateness and linguistic suitability. Translation accuracy and cultural resonance were assessed during the Cognitive Validity Index process, which involved evaluating both the linguistic precision and cultural appropriateness of survey items to ensure meaningful interpretation across common languages. For inclusion, each question must be reviewed by at least six experts and achieve a Cognitive Validity Index score above 0.8.^44^ Please see Table 3 and the Supplemental Material for detailed feedback on the Cognitive Validity Index scores.

**Table 3.** Cognitive Validity Index score for each question of the preliminary survey.

Item	Question	Expert 1	Expert 2	Expert 3	Expert 4	Expert 5	Expert 6	Expert 7	Cognitive Validity Index
1	**What is the name of your initiative?** *Free text answer*	3.25	3.75	3.5	2.75	3.5	2.5	4	0.71
2	**In what city and country is your initiative? (If it is online, please tell us that too!)** *Free text answer*	3.75	3	3.75	3.25	3.25	4	4	1.00
3	**What year did this initiative start?** *Drop down of years*	4	4	4	3.25	4	4	4	1.00
4	**What is your role within the organisation?** *(Unique choice answer)* - Paid staff member - Volunteer - Service user - Community member - Other: *free text*	3.75	3.75	3	4	3.25	4	3.5	1.00
4*	**What is your main role within the organisation?** (*Unique choice answer)* - Healthcare staff (e.g. physiotherapist, nurse, doctor) - Support with activities of daily living (e.g. providing support with personal care, e.g. washing, shopping) - Emotional support (e.g. counselling) - Community engagement (e.g. helping run community support groups) - Education or raising awareness - Spiritual support (e.g. a chaplain) - Administration (e.g. finances or documentation) - Policy development - Other: *free text*	3.75	3.75	3.5	4	3.5	4	3.75	1.00
5a	**How is this initiative funded?** *(Multiple choice answer)* Type of funding source: - Charity funding - Non-governmental organisations - Governmental funding - Healthcare commissioning (e.g. services are paid through the budget for the local hospital) - Donations - Other: *Free text*	3.5	4	3.5	4	4	4	3.5	1.00
5b	**How is this initiative funded?** *(Multiple choice answers)* Geographic funding source: - Local funding - National funding - International funding - Other: *free text*	3.75	4	4	4	4	3.75	3.5	1.00
6	**What are the main goals of this initiative?** (*Multiple choice*) - Support for people with chronic illness or at the end-of-life - Support for people who provide care (e.g. supporting family members caring for ill relatives, or professionals providing care) - Community empowerment (e.g. peer support groups) - Raising awareness about end-of-life or grief issues (often termed death literacy) - Policy (e.g. helping write guidelines to support those around the end-of-life) - Emotional/bereavement/grief support - Providing healthcare services such as pain relief - Other: *Free text*	3.5	3.75	4	4	2.75	3.75	3.25	0.86
7	**Who are the main people this initiative supports?** (e.g. age groups, patient populations, caregivers, health professionals, etc.) *Free text*	3.5	4	4	4	3	3.5	3.5	1.00
8	**How does your initiative reach these people or groups?** *(Multiple choice answer)* - Word of mouth - Referrals from healthcare providers - Advertising (e.g. flyers, newspapers) - Online advertising (e.g. social media posts, websites) - Community networks (e.g. local groups, organisations) - Social media platforms - Faith-based organisations (e.g. churches, temples, mosques) - Public awareness campaigns (e.g. health fairs, awareness weeks) - Government or municipal programme - School or university outreach - Workplace partnerships or employee assistance - Other: *Free text*	3.5	4	4	3.5	4	3.5	3.5	1.00
9	**Do you work with other organisations?** (*Multiple choice answer)* - Healthcare providers - No current collaboration with other organisations - Charities - Non-governmental organisations - Local or national governmental organisations - International organisations - Community-based organisations - Educational institutions (e.g. schools, universities) - Faith-based organisations (e.g. churches, temples, mosques) - Private companies or businesses - Cultural or arts organisations - Social service agencies - Other: *Free text*	3.75	4	3,5	4	3.75	4	3.5	1.00
10	**What outcomes have been seen from this work?** *Free text*	3.75	4	3.5	3	2.5	3.75	3.5	0.86
11	**How are the effects of this initiative measured?** (*Multiple choice answer)* - Effect measurement is not currently conducted for this programme - Feedback from participants or stakeholders - Number of people served - Growth in staff and volunteers involved - Staff and volunteer retention rates - Changes in healthcare resource utilisation - Economic evaluations (e.g. cost-effectiveness, cost savings) - Changes in community attitudes, behaviours and awareness of end-of-life matters - Collaborations with organisations or partners - Public recognition or awards received - Media coverage or mentions - Improved access and quality to end-of-life care resources - Influence on local or national policies - Other: *Free text*	3.75	4	3.75	3	4	3.5	3.75	1.00
12	**What challenges does this initiative face?** (*Multiple choice answer)* - Difficulty with growth, scaling and engaging communities - Financial challenges - Lack of awareness or visibility - Challenges in recruiting and keeping volunteers - Difficulty in maintaining a diverse and inclusive approach - Challenges working with healthcare providers - Difficulty in building long-term partnerships - Lack of support from local authorities, policymakers or legal/regulatory barriers - Limited data research to demonstrate the initiative impact - Cultural barriers or stigma around death and dying - Inadequate infrastructure or technology - Other: *Free text*	3.75	3.75	3.75	4	4	3.75	3.5	1.00
13	**What support would help this initiative grow?** *Free text*	3.75	4	3.75	4	4	3.75	3.5	1.00
14	**Were there any questions or options that seemed out of place? If you’re able to, can you explain why?** - Yes. Which ones: *Free text* - No	3.75	3.25	3.5	3.25	4	3	4	1.00
15	**Was there any topics or areas that are really important to your initiative that we did not ask about?** - Yes. Which ones: *Free text* - No	4	4	4	4	4	3	4	1.00

* Only if they answer to be paid staff.

The finalised set of questions was then imputed in Qualtrics, made accessible across different languages and devices, and a further round of testing was done. The survey questions and format were piloted again with both the core research team and the steering committee. Their final feedback was incorporated into the development of the definitive version of the survey.

### Data collection

The survey information will be displayed on social media, key partners websites, academic events in different regions (European, Asian-Pacific, African palliative care conferences and the research seminar on public health palliative care, to be held in Vancouver), blogs and through direct emails. It will also be disseminated to all members of existing networks and communities. Databases of existing compassionate communities compiled through literature, internet and social media searching, will also be used. The global steering committee, diverse networks and targeted dissemination aim to have global reach. Following dissemination through these initial channels, participants will be encouraged to share and recruit further participants, with the survey and relevant information available through QR codes and links. Reminder emails and posts will be sent, both to complete the survey and to encourage snowballing to other potential participants, with the survey open for 3 months. This method will allow the collection of large amounts of data, both geographically and numerically, in a timely manner.

### Analysis

The majority of the survey questions and potential responses have been designed with structured options to facilitate quantitative analysis. Descriptive content analysis will be applied to summarise and interpret the structured responses, focusing on key variables such as the geographical distribution of compassionate communities, the types of offered, the specific goals and objectives of these initiatives, sources of funding and the nature of the services provided. This will be used to provide a clear view of distribution and patterns across different respondents and characteristics to explore the variations and commonalities across different compassionate communities. The results will include categories and sub-analysis based on region, income level, type of programme and palliative care development stage to improve searchability and enable tailored analyses that can inform context-specific policy and program decisions.

Statistical analysis will be conducted using IBM SPSS Statistics V.30, where statistical tests and multivariate models will be applied to assess the distribution of responses and uncover any significant patterns or correlations between the various characteristics of the compassionate communities. These tests will allow for an examination of how different factors, such as geographical location, funding sources and programme objectives are distributed and how they interact.

For open-ended responses, we will perform descriptive analysis and explore patterns in the responses, such as self-reported goals, perceived impacts and challenges faced by each programme. NVivo will be used, and this approach will allow us to identify both key recurring themes and unique contributions across diverse contexts.^45–47^

As responses from citizens participating in the same compassionate communities will be collected based on their experiences and perspectives, contradictory or outlier data are possible. Reliability will be reviewed, including using the diverse steering group to promote translatability and cultural understanding of the findings.^40,48^

### Dissemination

A Virtual Atlas will display the key information about compassionate communities worldwide. Previous atlases, such as the Global Atlas of Palliative Care, the findings through the ATLANTES Global Observatory and regional atlases, have highlighted the strategic value of mapping, not only as a descriptive tool but as a way to raise awareness, enable benchmarking across contexts, highlight gaps and ultimately inform policy decisions.^35,49–51^ Mapping can make visible what is often invisible, especially in the context of social initiatives such as in community-based end-of-life care, thus becoming a powerful lever for advocacy and system-level change.^35,52^ This is why we have chosen to create an atlas; a freely available, dynamic resource that will be more accessible to a wide audience than traditional academic publications, promoting visibility and accessibility of information.^35,53^ We aim for the platform to be collaborative and not extractive, with voluntary contributions, valuing local perspectives and broad and inclusive representation rather than imposing rigid definitions. Moving away from expert-led models and embracing a participatory approach aims to allow Compassionate Communities themselves to shape how they are represented and guide our understanding in ways that reflect local realities. We will also publish a peer-reviewed manuscript to complement this broader, open-access resource.

### Results of the survey design and testing

A total of 18 researchers are involved in the design of the study and participation in the cognitive interviewing and survey piloting, originating from the following countries: Canada (*n* = 3), Australia (*n* = 3), United Kingdom (*n* = 2), Uganda (*n* = 2), Argentina, Belgium, Colombia, India, Japan, Rwanda, Spain and Thailand (*n* = 1 each). Ten participants (55%) were female. All have significant experience in public health palliative care. Fifteen participants (83%) were board members of national or international organisations engaged in research related to the implementation of community-based palliative care for both children and adult populations. Five researchers (L.S., S.V., V.T., J.E.C., R.N.) participated as part of the core research team and 13 as the steering research committee.

The core research team developed a preliminary version of the survey, which was subsequently reviewed by seven experts from the steering committee. Table 1 presents the mean scores for each question regarding clarity, relevance and cultural and linguistic appropriateness, along with the corresponding Content Validity Index (CVI) scores. Fourteen of the 15 questions met the established validity threshold. Individual expert scores and item-level CVI results are available in Supplemental File 1. Based on the detailed qualitative feedback, significant changes were made, including rephrasing and reordering survey items. Expert comments, along with the rationale and specific revisions, are documented in Supplemental File 2. This revised survey version was then built in Qualtrics and piloted with the 18 participating researchers. Feedback and subsequent amendments from this pilot phase are detailed in Supplemental File 3.

The final survey explores key characteristics of community-based that support individuals through illness, caregiving, dying and grief, gathering data on their structure, geographic location, activities and collaborative efforts. It also assesses how these initiatives measure their impact at individual and community levels, including service reach, policy influence and cultural or systemic change. Additionally, the survey captures perceived strengths and challenges, such as funding, visibility and community engagement. This offers insights into both the operational realities and strategic potential of these. It can be reviewed in its final version in Supplemental File 4.

## Discussion

Pain relief and palliative care are essential to achieving Universal Health Coverage, especially as the burden of serious, life-limiting illness and associated suffering continues to grow.^6,26^ Given the global need for more comprehensive palliative care coverage, the motivation to map and learn from compassionate communities lies in the potential of primary palliative care and compassionate communities as part of the solution.^5,12–14^ By engaging a diverse, global sample of social and civic related to death, dying and grief, this research aims to deliver a more representative and nuanced map of the type and variety of initiatives that exist, who they connect with and what outcomes they aim to achieve.

The atlas as a platform to connect communities and make their important work visible to the world aligns with the WHO’s global call to empower communities in developing primary palliative care by providing visibility to various initiatives, fostering opportunities for connection and facilitating mutual learning.^54,55^ Furthermore, this study builds on existing literature and incorporates lessons from previous research by addressing language barriers, employing broad inclusive frameworks and recruiting participants from diverse backgrounds and regions to overview and collect data. This will result in a more authentic and globally representative perspective.^56^

This study intentionally addresses a key limitation identified in prior global mapping efforts, namely the overreliance on expert opinion as the primary source of information. As highlighted by Loucka et al., such approaches risk introducing bias, limiting representation and failing to reflect the nuanced realities of palliative care on the ground.^57^ This risks reproducing models based on a small sample that may not work for most non-represented settings. In contrast, while our project benefited from expert input during the survey design phase, data collection itself enables individuals and communities to self-define compassionate communities. Furthermore, previous research aimed at identifying theoretical frameworks for evaluating the complex processes behind public health palliative care initiatives has found that while existing frameworks provide valuable insights into the dynamic interactions between contextual factors and the social processes shaping primary palliative care development and implementation, they are often incomplete, inflexible, insufficiently tested, and lack grounding in the lived experiences, needs and aspirations of communities.^13^ Our approach not only diversifies the voices included in the evidence base but also enhances validity by reducing single-informant bias and empowering those most closely engaged in compassionate community work to co-produce knowledge.^13,57^

However, it is important to remain aware of potential unintended consequences of mapping and raising awareness of these. As a research team, we risk perpetuating inequalities through our own frameworks and structures, meaning we only map those initiatives we already have models or understanding for, resulting in selectively promoting those initiatives that fit with our preconceived ideas. This will require ongoing reflection and active dialogue about our preconceptions and assumptions, continually asking who are we still excluding? Increased awareness and the potential implications of this such as formal funding or integration into existing services may unintentionally disrupt or ‘crowd out’ community-driven initiatives or take away procedural flexibility and creativity necessary to meet people’s needs. It can also create tensions between institutions, volunteer roles and social attitudes and ideals with regards to priorities, risk management and boundaries.^58^

Furthermore, while compassionate communities can offer vital support and compassion, they may also perpetuate exclusion, discrimination or harm. Determining responsibility for end-of-life care can also be complex; compassionate community initiatives, for instance, may inadvertently promote states or institutions to abdicate their responsibilities, undermining broader palliative care coverage.^59^ These frictions are rarely explored in the literature and for us to address them in a meaningful way, it is essential that we understand the reality of compassionate communities from a range of voices and contexts.^59^

In recognition of these risks, we have designed the atlas as a voluntary, community-led initiative in which communities self-report their characteristics and aims, outcomes and challenges. This approach aims to reduce the risk of institutional co-option and allow communities to retain ownership and control over their narratives. While it may not be possible to prevent all unintended consequences, we are committed to ongoing dialogue, transparency and responsiveness as the project develops.

There are inherent limitations to this survey and its methodology. Reliance on self-reported data may mean errors exist such as the over-reporting of outcomes or objectives. Given the complexity, diversity and often informal nature of compassionate communities, no single tool can fully capture their richness or nuance. The survey may overlook or misinterpret key contextual elements, and it is likely that some initiatives will remain undocumented due to language barriers, digital access or differing understandings of what constitutes a compassionate community. Although translation and cultural interpretation were assessed for common languages during the validation process, this was not possible for all languages and remains a limitation. Communities without reliable digital infrastructure, translation support or formal recognition may remain underrepresented. Additionally, those with closer proximity to academic or policy networks may have greater visibility, perpetuating existing inequities.

These factors will introduce sampling bias, and we acknowledge that this; alongside unintended exclusion, especially of informal or undocumented groups, remains a limitation; however, the Atlas may help identify these gaps and guide future research to better include these communities. The final question of the survey will ask for comments on areas the survey did not cover, with the hope that this could be a first iteration, to be built upon as we expand our understanding of compassionate communities globally.

We are aiming for the atlas to be a sustainable resource, with future iterations and ongoing updates. While this research project focuses on the initial development and data collection, the Atlas will be hosted by PHPCI. Subject to future engagement and available resources, we hope to conduct follow-up surveys and we also aim to enable communities to expand their entries with additional information, such as website links or descriptive content. This aims to enable communities to strengthen their work locally, while also feeling connected to and recognised within the broader global movement around community-based palliative care. Through this approach, we seek to maintain the Atlas as a community-led resource that supports continued participation, consent and control beyond the scope of this study.

We also hope this resource will help inform policy and funding decisions related to Primary Palliative Care and Universal Health Coverage by highlighting the role and global reach of Compassionate Communities and facilitating dialogue between different actors. This aligns with the WHO’s emphasis on community empowerment and integrated people-centred care.

Furthermore, while the survey allows for broad participation, it cannot provide the depth of insight that qualitative methods; such as in-depth interviews or ethnographic studies, could offer.^60^ Only including text-based responses, is a limitation, excluding the diverse forms of community knowledge, such as oral and visual histories. These methods would be invaluable in exploring the lived experiences, cultural dimensions and unique innovations that communities bring to responses around death, dying, grief and loss.

Nonetheless, we propose this survey as a step towards building a more comprehensive understanding of global public health palliative care efforts. The resulting atlas can serve not only as a resource in its own right but also as a more representative sampling frame for future, deeper qualitative or mixed-methods research.

## Conclusion

Compassionate communities do not have a single, static structure but are instead shaped by the specific cultural, social and political contexts in which they operate.^11,13,14,30^ Their true richness lies in the creativity of the communities themselves. The global atlas and methodology proposed in this protocol seek to reflect this contextual richness and aim to move towards a decolonised understanding of community programme addressing serious illness, death, dying and grief. By offering visibility to diverse community-based initiatives, the atlas aims to advance current knowledge, contribute to a more inclusive global evidence base and foster inclusive public health palliative care policies. While imperfect, this effort begins to fill a critical gap in the field by highlighting compassionate communities and the care they provide, and by offering a platform for future collaborative research.

## Supplemental Material

sj-docx-4-pcr-10.1177_26323524251396994 – Supplemental material for Connecting communities across the globe: Atlas protocol Supplemental material, sj-docx-4-pcr-10.1177_26323524251396994 for Connecting communities across the globe: Atlas protocol by Rebecca Newell, Juan Esteban Correa-Morales, Vilma A. Tripodoro, Steven Vanderstichelen, Ghauri Aggarwal, Samar Aoun, Erin Das, Farah Demachkieh, James Downar, Silvia Librada, Julieanne Hilbers, Julie Lapenskie, Emmanuel Luyirika, Saif Mohammed, Masanori Mori, Ekkapop Sittiwantana and Libby Sallnow in Palliative Care and Social Practice

sj-xlsx-1-pcr-10.1177_26323524251396994 – Supplemental material for Connecting communities across the globe: Atlas protocol Supplemental material, sj-xlsx-1-pcr-10.1177_26323524251396994 for Connecting communities across the globe: Atlas protocol by Rebecca Newell, Juan Esteban Correa-Morales, Vilma A. Tripodoro, Steven Vanderstichelen, Ghauri Aggarwal, Samar Aoun, Erin Das, Farah Demachkieh, James Downar, Silvia Librada, Julieanne Hilbers, Julie Lapenskie, Emmanuel Luyirika, Saif Mohammed, Masanori Mori, Ekkapop Sittiwantana and Libby Sallnow in Palliative Care and Social Practice

sj-xlsx-2-pcr-10.1177_26323524251396994 – Supplemental material for Connecting communities across the globe: Atlas protocol Supplemental material, sj-xlsx-2-pcr-10.1177_26323524251396994 for Connecting communities across the globe: Atlas protocol by Rebecca Newell, Juan Esteban Correa-Morales, Vilma A. Tripodoro, Steven Vanderstichelen, Ghauri Aggarwal, Samar Aoun, Erin Das, Farah Demachkieh, James Downar, Silvia Librada, Julieanne Hilbers, Julie Lapenskie, Emmanuel Luyirika, Saif Mohammed, Masanori Mori, Ekkapop Sittiwantana and Libby Sallnow in Palliative Care and Social Practice

sj-xlsx-3-pcr-10.1177_26323524251396994 – Supplemental material for Connecting communities across the globe: Atlas protocol Supplemental material, sj-xlsx-3-pcr-10.1177_26323524251396994 for Connecting communities across the globe: Atlas protocol by Rebecca Newell, Juan Esteban Correa-Morales, Vilma A. Tripodoro, Steven Vanderstichelen, Ghauri Aggarwal, Samar Aoun, Erin Das, Farah Demachkieh, James Downar, Silvia Librada, Julieanne Hilbers, Julie Lapenskie, Emmanuel Luyirika, Saif Mohammed, Masanori Mori, Ekkapop Sittiwantana and Libby Sallnow in Palliative Care and Social Practice
