# Supplementary material for: Connecting communities across the globe: Atlas protocol
Source: Palliat Care Soc Pract. 2025 Dec 4;19:26323524251396994. doi: 10.1177/26323524251396994 (PMC12681578; doi:10.1177/26323524251396994)
Supplement: sj-docx-4-pcr-10.1177_26323524251396994 – Supplemental material for Connecting communities across the globe: Atlas protocol [file sj-docx-4-pcr-10.1177_26323524251396994.docx]

Connecting Communities Across the Globe

Start of Block:

| 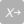 |
| --- |

**Informed Consent**   We invite you to take part in a global survey exploring community-based initiatives that support people through illness, caregiving, dying, and grief — often referred to as compassionate communities.  A community program is any organised effort within a community that helps people with experiences of illness, caregiving, death, dying, and grief. These programs can take many forms, such as support groups, awareness campaigns, or educational programs.   What is the Purpose of This Survey?

 Learn where these community programs exist Understand how they work and what they do Build a Global Atlas of Communities Programs  The atlas will raise awareness, encourage support, and help communities around the world share ideas and learn from each other.   Who Should Complete This Survey?
 A community member A volunteer A staff member Someone who knows about or has received support from the program   You will answer based on your own experience, not as an official representative of the program. More than one person from the same program can respond. Please note: We may not include your response in the atlas if it is not related to a community program addressing illness, dying, caregiving, and grief.   Voluntary Participation
 The survey will take around 10 minutes. Participation is completely voluntary. You may skip any question or stop at any time. After finishing, you will click to submit the survey and then will receive a code that allows you to withdraw your answers within four weeks with no reason needed.                                          Language options: You can translate this survey at the top right or by using your browser extension and picking the translate option. Confidentiality   No personal or identifying information will be collected. Your answers will stay anonymous and secure. The name and location of the community program may be shown in the atlas. Other responses will not be shared publicly. Anonymous data may be shared with other researchers for future studies. All data will be deleted by the end of 2026.   Risks and Benefits   There are no major risks to taking part. Some questions may bring up strong emotions, especially around illness and grief. You are free to skip any questions or stop the survey at any time. Only the location and name of the community program will be listed on the Global Atlas, which will be available on the Public Health Palliative Care International website.   Contact Information
 If you have any questions about the survey or your rights as a participant, please contact: Juan Esteban Correa at juan.correa@ucl.ac.uk or (+44) 7907 433051 Libby Sallnow at l.sallnow@ucl.ac.uk Or the UCL Ethics Committee at ethics@ucl.ac.uk

- I confirm that I am over 18 years old, have read and understood the information above, and give my consent to take part in this study. (1)

| Page Break |  |
| --- | --- |

**What is the name of your community program?** A community program is any organised effort within a community to focus on caregiving, death, dying and grief.

________________________________________________________________

| Page Break |  |
| --- | --- |

| 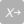 |
| --- |

In what country is your program?

▼ Afghanistan (1) ... Vatican City (197)

What is the location of your program? (Please provide local area)

________________________________________________________________

| Page Break |  |
| --- | --- |

| 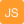 | 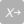 |
| --- | --- |

When did this program start?

|  | Year |
| --- | --- |
|  |  |
| Please Select: (1) | ▼ 1980 or earlier (1 ... 2025 (46) |

| Page Break |  |
| --- | --- |

| 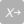 |
| --- |

What is your main role within this program?

- Volunteer (1)
- Community member (2)
- Healthcare professional (e.g. nurse, doctor, social worker, psychologist) (3)
- Administration (e.g. funding or staffing) (4)
- Senior staff / Board members (5)
- Spiritual support (e.g. chaplain) (6)
- Other: (7) __________________________________________________

Display this question:

If What is your main role within this program? = Healthcare professional (e.g. nurse, doctor, social worker, psychologist)

If you answered "healthcare worker" please select the type of healthcare worker.

- Nurse (1)
- Doctor (2)
- Community healthcare worker (3)
- Social worker (4)
- Psychologist (5)
- Psychiatrist (6)
- Councillor (7)
- Traditional healer (8)
- Other (9) __________________________________________________

Display this question:

If What is your main role within this program? = Volunteer

Or What is your main role within this program? = Community member

If you answered "volunteer" or "community member" please tell us about the types of responsibilities you have.

- Direct care (e.g.providing support with washing) (1)
- Emotional support (2)
- Education or training (4)
- Community engagement (5)
- Other (6) __________________________________________________

| Page Break |  |
| --- | --- |

| 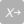 |
| --- |

What are the main activities of this program? Please select all that apply.

- Providing healthcare services such as pain relief (1)
- Support for people with chronic illness or at the end of life (2)
- Training and support for healthcare providers (3)
- Training and support for local leaders (4)
- Training and support for family, friends or local communities providing care (5)
- Raising awareness about end of life or grief issues (often termed death literacy) (6)
- Policy advocacy (e.g. helping write guidelines to support those around the end of life) (7)
- Bereavement / grief support (8)
- Generating / supporting community networks (9)
- Develop research (10)
- Support for underserved or vulnerable populations. Please specifiy which populations: (11) __________________________________________________
- Other: (12) __________________________________________________

If you would like to share your mission statement, values, or further information share it below:

________________________________________________________________

________________________________________________________________

________________________________________________________________

________________________________________________________________

________________________________________________________________

| Page Break |  |
| --- | --- |

| 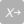 |
| --- |

What outcomes of this program are measured?

- Outcomes are not currently measured for this program (1)
- Service reach and use (e.g. number of people served) (3)
- Impact on individuals (e.g. reduced loneliness) (4)
- Impact on communities (e.g. community education) (5)
- Workforce and program development (e.g. staff development) (7)
- Recognition, policy and influence (8)
- Other: (20) __________________________________________________

Display this question:

If What outcomes of this program are measured? = Service reach and use (e.g. number of people served)

| 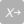 |
| --- |

 If you selected "Service reach and use" please specify the outcomes measures. (Select all that apply)
 Please select one of the following for Recognition, Policy, and Influence: Public recognition or awards Media coverage or mentions Influence on local or national policies

- Number of people reached (3)
- Healthcare use that aligns with the wishes of those at the end of life and their loved ones (9)
- Enhance support for individuals and communities around end of life matters (10)
- Improved access and quality to end of life care resources (13)
- Other (14) __________________________________________________

Display this question:

If What outcomes of this program are measured? = Impact on individuals (e.g. reduced loneliness)

If you selected "Impact on individuals" please specify the outcomes measures. (Select all that apply)

- Patient-reported outcomes (e.g. physical or spiritual symptoms) (1)
- Patient or career satisfaction (7)
- Improved social support around end-of-life (2)
- Addressing living conditions for people requiring care (4)
- Other (5) __________________________________________________

Display this question:

If What outcomes of this program are measured? = Impact on communities (e.g. community education)

If you selected "Impact on communities" please specify the outcomes measures. (Select all that apply)
 Please select one of the following for Recognition, Policy, and Influence: Public recognition or awards Media coverage or mentions Influence on local or national policies

- Reduction in health inequalities among underserved populations (1)
- Strengthening capacity of communities to manage end-of-life matters (2)
- Changes in community attitudes, behaviours or awareness around end-of-life (3)
- Enhanced support for communities around end-of-life (4)
- Other (5) __________________________________________________

Display this question:

If What outcomes of this program are measured? = Recognition, policy and influence

If you selected "Recognition, policy and influence" please specify the outcomes measures. (Select all that apply)
 Please select one of the following for Recognition, Policy, and Influence: Public recognition or awards Media coverage or mentions Influence on local or national policies

- Public recognition or awards received (1)
- Media coverage or public mentions (2)
- Influence on local or national policies (3)
- Other (4) __________________________________________________

Display this question:

If What outcomes of this program are measured? = Workforce and program development (e.g. staff development)

If you selected "Workforce and program development " please specify the outcomes measures. (Select all that apply)

- Engagement, development, and retention of staff and volunteers (1)
- Collaborations with other organizations or partners (2)
- Research output and productivity (e.g. reports, articles, scientific publications) (3)
- Other (4) __________________________________________________

| Page Break |  |
| --- | --- |

| 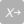 |
| --- |

How do people find their way to your program? Please select all that apply.

- Word of mouth (1)
- Referrals from healthcare providers (2)
- Advertising (e.g. flyers, newspapers) (3)
- Online Advertising (e.g. social media posts, websites) (4)
- Community networks (e.g. local groups, organizations) (5)
- Faith-based organizations (e.g. churches, temples, mosques) (6)
- Public awareness campaigns (e.g. health fairs, awareness weeks) (7)
- Government or municipal programs (8)
- Educational institutions ( e.g. schools, universities) (9)
- Workplace partnerships or employee assistance programs (10)
- Other: (11) __________________________________________________

| Page Break |  |
| --- | --- |

| 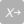 |
| --- |

Which of the following organizations has this project collaborated with? Please select all that apply.

- No current collaboration with other organizations (1)
- Healthcare organizations / associations (2)
- Governmental organizations / associations (6)
- Social service or support agencies (13)
- Charities (3)
- Patients organizations / associations (5)
- Community-based organizations / associations (8)
- Faith-based organizations (e.g. churches, temples, mosques) (10)
- Cultural or arts organizations / associations (12)
- Educational institutions (e.g. schools, universities) (9)
- Private companies or businesses (11)
- I dont know this information (14)
- Other: (15) __________________________________________________

Display this question:

If Which of the following organizations has this project collaborated with? Please select all that a... = Healthcare organizations / associations

Or Which of the following organizations has this project collaborated with? Please select all that a... = Charities

Or Which of the following organizations has this project collaborated with? Please select all that a... =

Or Which of the following organizations has this project collaborated with? Please select all that a... = Patients organizations / associations

Or Which of the following organizations has this project collaborated with? Please select all that a... = Governmental organizations / associations

Or Which of the following organizations has this project collaborated with? Please select all that a... =

Or Which of the following organizations has this project collaborated with? Please select all that a... = Community-based organizations / associations

Or Which of the following organizations has this project collaborated with? Please select all that a... = Educational institutions (e.g. schools, universities)

Or Which of the following organizations has this project collaborated with? Please select all that a... = Faith-based organizations (e.g. churches, temples, mosques)

Or Which of the following organizations has this project collaborated with? Please select all that a... = Private companies or businesses

Or Which of the following organizations has this project collaborated with? Please select all that a... = Cultural or arts organizations / associations

Or Which of the following organizations has this project collaborated with? Please select all that a... = Social service or support agencies

What are the aims of the collaborations?

- Providing patient care (1)
- Raising awareness (2)
- Strengthening support networks (3)
- Influencing Policy (4)
- Education and Training (5)
- Bridging gaps in care or support (6)
- Other (7) __________________________________________________
- If you would like to tell us more about collaborations please do so below: (8) __________________________________________________

| Page Break |  |
| --- | --- |

| 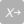 |
| --- |

How is this program funded? Please select all that apply.

- Public/ governmental funding (1)
- Private funding (5)
- I dont know this information (12)
- No current funding being received (14)
- Other: (13) __________________________________________________

Display this question:

If How is this program funded? Please select all that apply. = Private funding

| 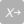 |
| --- |

If you selected "Private funding," please specify the sources of funding. (Select all that apply)

- Charity funding (1)
- Non-Governmental Organizations (NGOs) (2)
- Out-of-pocket expenses (costs you have to pay yourself, not covered by insurance or other sources) (6)
- Funding through membership fees (7)
- Donations (9)
- Insurance (10)
- Non-monetary goods (11)
- Other: (13) __________________________________________________

Display this question:

If How is this program funded? Please select all that apply. = Public/ governmental funding

| 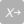 |
| --- |

If you selected "Public funding," please specify the sources of funding. (Select all that apply)

- Governmental funding (1)
- Grants (2)
- Healthcare budget (e.g. services are paid through the budget for the hospital) (6)
- Other: (13) __________________________________________________

| Page Break |  |
| --- | --- |

| 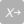 |
| --- |

For each of the following tell us if this is a threat or a strengths for your program:

|  | Threat (1) | Neutral (2) | Strength (4) |
| --- | --- | --- | --- |
| Scaling the program (1) |  |  |  |
| Engaging with communities (2) |  |  |  |
| Financial stability (3) |  |  |  |
| Awareness or visibility (4) |  |  |  |
| Recruiting and keeping volunteers and staff (6) |  |  |  |
| Local healthcare resources (8) |  |  |  |
| Support from local authorities or policymakers (18) |  |  |  |
| Data research or technology tools to demonstrate impact (19) |  |  |  |
| Cultural aspects around death and dying (21) |  |  |  |
| Distance and rurality (24) |  |  |  |
| Other community programs (29) |  |  |  |

Please write below if you would like to tell us more about threats or strengths to your program.

________________________________________________________________

________________________________________________________________

________________________________________________________________

________________________________________________________________

________________________________________________________________

| Page Break |  |
| --- | --- |

| 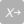 |
| --- |

Are there any important topics about your program that we didn't ask about but you would like to mention?

- Yes. Which ones: (1) __________________________________________________
- No (2)

| Page Break |  |
| --- | --- |

End of Block:

Start of Block:

Here is your unique completion code: ${rand://int/10000:99999} Please copy this number. Once you've copied your completion code, click Submit to finish the survey.

End of Block:
